# Supplementary material for: Establishment of a neonatal resuscitation registry in the Democratic Republic of the Congo: An open cohort study
Source: PLoS One. 2025 May 23;20(5):e0324332. doi: 10.1371/journal.pone.0324332 (PMC12101705; doi:10.1371/journal.pone.0324332)
Supplement: S1 Table — (DOCX) [file pone.0324332.s003.docx]

**Supplemental Table 1: Maternal and neonatal characteristics observed and not observed with the Liveborn Observation app**

| **Demographics**  **n (%)** | **Observed with Liveborn**  **N=3,166** | **Not observed with Liveborn**  **N=3,248** |  |
| --- | --- | --- | --- |
| **Maternal age in years** |  |  |  |
| < 20 | 116 (3.7%) | 158 (4.9%) |  |
| 20 – 35 | 2532 (80%) | 2567 (79%) |  |
| > 35 | 518 (16.4%) | 523 (16.1%) |  |
| Unknown | 0 (0%) | 0 (0%) |  |
| **Maternal parity** |  |  |  |
| 0 | 793 (25%) | 887 (27.3%) |  |
| 1-2 | 1466 (46.3%) | 1372 (42.2%) |  |
| ≥ 3 | 906 (28.6%) | 989 (30.4%) |  |
| Unknown | 1 (0%) | 0 (0%) |  |
| **Mode of delivery** |  |  |  |
| Spontaneous vaginal | 3149 (99.5%) | 3175 (97.8%) |  |
| Vaginal with forceps/vacuum | 9 (0.3%) | 3 (0.1%) |  |
| Cesarean section | 8 (0.3%) | 70 (2.2%) |  |
| Unknown | 0 (0%) | 0 (0%) |  |
| **Birth weight in grams** |  |  |  |
| ≤ 1499 | 33 (1%) | 105 (3.2%) |  |
| 1500 – 2499 | 293 (9.3%) | 416 (12.8%) |  |
| ≥ 2500 | 2840 (89.7%) | 2725 (83.9%) |  |
| Unknown | 0 (0%) | 2 (0.1%) |  |
| **Gestational age in weeks** |  |  |  |
| < 28 | 6 (0.2%) | 7 (0.2%) |  |
| 28 - 36 | 396 (12.5%) | 582 (17.9%) |  |
| ≥ 37 | 2744 (86.7%) | 2647 (81.5%) |  |
| Unknown | 20 (0.6%) | 12 (0.4%) |  |
| **Newborn sex** |  |  |  |
| Male | 1654 (52.2%) | 1646 (50.7%) |  |
| Female | 1512 (47.8%) | 1601 (49.3%) |  |
| Unknown | 0 (0%) | 1 (0%) |  |
| **Congenital anomalies** | (0%) | (0%) |  |
| Any malformation | 4 (0.1%) | 6 (0.2%) |  |
| Neural tube defect | 0 (0%) | 0 (0%) |  |
| Abdominal wall defect | 0 (0%) | 1 (16.7%) |  |
| Other | 4 (100%) | 6 (100%) |  |
| No malformation | 3162 (99.9%) | 3241 (99.8%) |  |
| Unknown | 0 (0%) | 1 (0%) |  |
| **Multiplicity** |  |  |  |
| Singleton | 3091 (97.6%) | 3152 (97%) |  |
| Twin | 74 (2.3%) | 93 (2.9%) |  |
| Multiple beyond twins | 1 (0%) | 3 (0.1%) |  |
| Unknown | 0 (0%) | 0 (0%) |  |
| **APGAR scores** |  |  |  |
| APGAR 1min ≤5 | | 148 (4.7%) | 239 (7.4%) |
| Unknown | | 0 (0%) | 0 (0%) |
| APGAR 5min ≤5 | | 42 (1.3%) | 107 (3.3%) |
| Unknown | | 0 (0%) | 0 (0%) |
| **Vital status at discharge** |  |  |  |
| Stillbirth | 29 (0.9%) | 97 (3%) |  |
| Fresh stillbirth | 13 (0.4%) | 49 (1.5%) |  |
| Macerated stillbirth | 16 (0.5%) | 47 (1.4%) |  |
| Unknown | 0 (0%) | 1 (0%) |  |
| Neonatal death before discharge | 27 (0.9%) | 64 (2%) |  |
| Transferred | 44 (1.4%) | 37 (1.1%) |  |
| Alive at discharge | 3063 (96.7%) | 3049 (93.9%) |  |
| Unknown | 3 (0.1%) | 1 (0%) |  |
