# Supplementary material for: Establishment of a neonatal resuscitation registry in the Democratic Republic of the Congo: An open cohort study
Source: PLoS One. 2025 May 23;20(5):e0324332. doi: 10.1371/journal.pone.0324332 (PMC12101705; doi:10.1371/journal.pone.0324332)
Supplement: S2. Table — (DOCX) [file pone.0324332.s004.docx]

**Supplemental Table 2:** **Maternal and neonatal characteristics cumulative and divided by facility**

| **Demographics**  **n (%)** | **Cumulative**  **N=6,414** | **Facility 1**  **N=3,414** | **Facility 2**  **N=3,000** |
| --- | --- | --- | --- |
| **Maternal age in years** |  |  |  |
| < 20 | 274 (4.3%) | 113 (3.3%) | 161 (5.4%) |
| 20 – 35 | 5099 (79.5%) | 2780 (81.4%) | 2319 (77.3%) |
| > 35 | 1041 (16.2%) | 521 (15.3%) | 520 (17.3%) |
| Unknown | 0 (0%) | 0 (0%) | 0 (0%) |
| **Maternal parity** |  |  |  |
| 0 | 1680 (26.2%) | 924 (27.1%) | 756 (25.2%) |
| 1-2 | 2838 (44.2%) | 1568 (45.9%) | 1270 (42.3%) |
| ≥ 3 | 1895 (29.5%) | 921 (27%) | 974 (32.5%) |
| Unknown | 1 (0%) | 1 (0%) | 0 (0%) |
| **Mode of delivery** |  |  |  |
| Spontaneous vaginal | 6324 (98.6%) | 3413 (100%) | 2911 (97%) |
| Vaginal with forceps/vacuum | 12 (0.2%) | 1 (0%) | 11 (0.4%) |
| Cesarean section | 78 (1.2%) | 0 (0%) | 78 (2.6%) |
| Unknown | 0 (0%) | 0 (0%) | 0 (0%) |
| **Birth weight in grams** |  |  |  |
| ≤ 1499 | 138 (2.2%) | 13 (0.4%) | 125 (4.2%) |
| 1500 – 2499 | 709 (11.1%) | 302 (8.8%) | 407 (13.6%) |
| ≥ 2500 | 5565 (86.8%) | 3097 (90.7%) | 2468 (82.3%) |
| Unknown | 2 (0%) | 2 (0.1%) | 0 (0%) |
| **Gestational age in weeks** |  |  |  |
| < 28 | 13 (0.2%) | 3 (0.1%) | 10 (0.3%) |
| 28 - 36 | 978 (15.2%) | 375 (11%) | 603 (20.1%) |
| ≥ 37 | 5391 (84.1%) | 3004 (88%) | 2387 (79.6%) |
| Unknown | 32 (0.5%) | 32 (0.9%) | 0 (0%) |
| **Newborn sex** |  |  |  |
| Male | 3300 (51.4%) | 1735 (50.8%) | 1565 (52.2%) |
| Female | 3113 (48.5%) | 1679 (49.2%) | 1434 (47.8%) |
| Unknown | 1 (0%) | 0 (0%) | 1 (0%) |
| **Congenital anomalies** |  |  |  |
| Any malformation | 10 (0.2%) | 5 (0.1%) | 5 (0.2%) |
| Neural tube defect | 0 (0%) | 0 (0%) | 0 (0%) |
| Abdominal wall defect | 1 (10%) | 1 (20%) | 0 (0%) |
| Other | 10 (100%) | 5 (100%) | 5 (100%) |
| No malformation | 6403 (99.8%) | 3408 (99.8%) | 2995 (99.8%) |
| Unknown | 1 (0%) | 1 (0%) | 0 (0%) |
| **Multiplicity** |  |  |  |
| Singleton | 6243 (97.3%) | 3341 (97.9%) | 2902 (96.7%) |
| Twin | 167 (2.6%) | 72 (2.1%) | 95 (3.2%) |
| Multiple beyond twins | 4 (0.1%) | 1 (0%) | 3 (0.1%) |
| Unknown | 0 (0%) | 0 (0%) | 0 (0%) |
| **APGAR scores** |  |  |  |
| APGAR 1min ≤5 | 387 (6%) | 133 (3.9%) | 254 (8.5%) |
| Unknown | 0 (0%) | 0 (0%) | 0 (0%) |
| APGAR 5min ≤5 | 149 (2.3%) | 57 (1.7%) | 92 (3.1%) |
| Unknown | 0 (0%) | 0 (0%) | 0 (0%) |
| **Vital status at discharge** |  |  |  |
| Stillbirth | 126 (2%) | 39 (1.1%) | 87 (2.9%) |
| Fresh stillbirth | 62 (1%) | 22 (0.6%) | 40 (1.3%) |
| Macerated stillbirth | 63 (1%) | 16 (0.5%) | 47 (1.6%) |
| Unknown | 1 (0%) | 1 (0%) | 0 (0%) |
| Neonatal death before discharge | 91 (1.4%) | 21 (0.6%) | 70 (2.3%) |
| Transferred | 81 (1.3%) | 71 (2.1%) | 10 (0.3%) |
| Alive at discharge | 6112 (95.3%) | 3279 (96%) | 2833 (94.4%) |
| Unknown | 4 (0.1%) | 4 (0.1%) | 0 (0%) |
