# Supplementary material for: Establishment of a neonatal resuscitation registry in the Democratic Republic of the Congo: An open cohort study
Source: PLoS One. 2025 May 23;20(5):e0324332. doi: 10.1371/journal.pone.0324332 (PMC12101705; doi:10.1371/journal.pone.0324332)
Supplement: Attachment — Submitted filename: Response to Reviewers.pdf [file pone.0324332.s007.pdf]

UNC SCHOOL OF MEDICINE

UNC HEALTH Children's

Division of Neonatal-Perinatal Medicine
The University of North Carolina at Chapel Hill
UNC Hospitals, CB# 7596, 4th Floor, Rm N4051
Chapel Hill, NC 27599-7596
Office: 984-974-5063 • Fax: 984-974-7857

January 24, 2025

Sebastian Schnaubelt, MD, PhD
Academic Editor
PLOS ONE

Dear Dr. Schnaubelt,

Thank you for your continued consideration of our manuscript, "Establishment of a neonatal resuscitation registry in the Democratic Republic of the Congo: An open cohort study" (*PONE-D-24-30580*). Following is a point-by-point response to the reviewer's comments to match the revised manuscript we have submitted.

Sincerely,

Jackie K. Patterson, MD, MPH
Associate Professor of Pediatrics
Division of Neonatal-Perinatal Medicine
University of North Carolina at Chapel Hill

UNC SCHOOL OF MEDICINE

UNC HEALTH Children's

Division of Neonatal-Perinatal Medicine
The University of North Carolina at Chapel Hill
UNC Hospitals, CB# 7596, 4th Floor, Rm N4051
Chapel Hill, NC 27599-7596
Office: 984-974-5063 • Fax: 984-974-7857

Reviewer 2 Feedback:

1) Introduce a comparative analysis where possible, or discuss the limitations of the study design regarding the absence of a control group. This could help contextualize the findings within the broader literature on neonatal resuscitation practices.

Response:
Our access to a control group would be historically remote (using data from a former newborn resuscitation trial in these facilities conducted a few years prior) or retrospective (using medical record abstraction with little data on actual resuscitation care). As such, in our judgement, the potential comparators are not robust enough to draw conclusions. To address the lack of a comparator group, we added a sentence to the limitations section of the discussion:

> "The study design did not include comparative analysis, which limits our understanding of the effect of establishing the registry." (lines 349-350)

Of note, the third paragraph of the discussion section includes a comparison of our findings to our prior study in the DRC as well as prior literature from Tanzania and Uganda (lines 265-268).

2) Expand the discussion on the implications of registry findings for neonatal care practices and policy, both within the DRC and globally. Highlighting specific variables that were not collected but are critical for improving neonatal outcomes could be particularly insightful.

Response:
We expanded the discussion on implications of registry findings with attention to our findings of excessive suctioning and interruptions in PPV (lines 268-269), the subjectivity of evaluation of breathing (lines 300-302) and the need to expand the target population for evaluating quality resuscitation (lines 322-326). We also highlighted the lack of intrapartum data currently collected in the registry and the importance of addressing the intrapartum period to improve neonatal outcomes (lines 343-344).

3) Streamline the introductory and methodology sections to maintain a sharper focus on the study's objectives, findings, and implications. Reducing descriptive content that does not directly contribute to understanding the study's impact could enhance readability and engagement.

Response:
We revised the introductory and methodology sections per your suggestion to be more succinct and maintain a sharper focus.

UNC SCHOOL OF MEDICINE

UNC HEALTH Children's

Division of Neonatal-Perinatal Medicine
The University of North Carolina at Chapel Hill
UNC Hospitals, CB# 7596, 4th Floor, Rm N4051
Chapel Hill, NC 27599-7596
Office: 984-974-5063 • Fax: 984-974-7857

4) Provide a more detailed analysis of the barriers and facilitators encountered during the registry's implementation, including how these challenges were addressed or could be overcome in future research. Sharing lessons learned in this aspect could be extremely valuable to others looking to implement similar registries in low-resource settings.

Response:
We re-organized the results section to cluster our analysis of barriers and facilitators in one paragraph, and bolstered this analysis with more detail regarding lessons learned (lines 183-199). We also added more detail to the discussion section regarding the burden of paper record-keeping (lines 246-249), the challenges around midwives consistently using NeoBeat (lines 352-355) and the resources involved in adding research staff to conduct observations with Liveborn (lines 361-362).

Reviewer 3 comments:

1) It is crucial that feedback is provided to the ILCOR neonatal group so that any revision of the Utstein criteria can be updated whenever appropriate. It is however "the hope of the Neonatal Utstein Working Group that, at minimum, all specified core data elements will be reported using the standardized definitions provided here." It would be useful to provide feedback not only about what data was difficult to collect, but in what way (if any) the authors planned to explore collecting more of the core data elements. Barriers to collection are crucial to discuss and solutions may need to be creative. If this is not possible, it may be that the key components of this manuscript would be better published as a letter.

Response:
We added more detail regarding challenges with collecting certain data elements to the results section, with a focus on core data elements (lines 183-199; see response to #4 from reviewer 2). We also highlighted the new core variables we have since added to the registry in the discussion (lines 246-253).
