# Supplementary material for: Establishment of a neonatal resuscitation registry in the Democratic Republic of the Congo: An open cohort study
Source: PLoS One. 2025 May 23;20(5):e0324332. doi: 10.1371/journal.pone.0324332 (PMC12101705; doi:10.1371/journal.pone.0324332)
Supplement: Attachment — Submitted filename: Response to Reviewers 4-9-25.pdf [file pone.0324332.s008.pdf]

UNC SCHOOL OF MEDICINE

UNC HEALTH Children's

Division of Neonatal-Perinatal Medicine
The University of North Carolina at Chapel Hill
UNC Hospitals, CB# 7596, 4th Floor, Rm N4051
Chapel Hill, NC 27599-7596
Office: 984-974-5063 • Fax: 984-974-7857

April 9, 2025

Sebastian Schnaubelt, MD, PhD
Academic Editor
PLOS ONE

Dear Dr. Schnaubelt,

Thank you for your continued consideration of our manuscript, "Establishment of a neonatal resuscitation registry in the Democratic Republic of the Congo: An open cohort study" (*PONE-D-24-30580*). Following is a point-by-point response to the reviewer's comments to match the revised manuscript we have submitted.

Sincerely,

Jackie K. Patterson, MD, MPH
Associate Professor of Pediatrics
Division of Neonatal-Perinatal Medicine
University of North Carolina at Chapel Hill

UNC SCHOOL OF MEDICINE

UNC HEALTH Children's

Division of Neonatal-Perinatal Medicine
The University of North Carolina at Chapel Hill
UNC Hospitals, CB# 7596, 4th Floor, Rm N4051
Chapel Hill, NC 27599-7596
Office: 984-974-5063 • Fax: 984-974-7857

Reviewer 4 Feedback:

Methods
1. Were there any quality checks or inter-rater reliability checks given the diversity of background of raters (midwives, doctors, etc)?

Response:
We did not perform quality checks or inter-rater reliability of the observations. We added a sentence explicitly stating this in the limitations of the discussion (lines 377-379).

Of note, in the discussion, we detailed that Liveborn Observation app registrations were likely delayed and that observers with clinical responsibilities may have competing tasks; thus, we began restricting data collection to study nurses only. In the future directions in the last paragraph of the discussion, we mentioned our plans to validate the accuracy of observational data compared to audio-video recording, which is currently underway.

2. How were you handling missing data?

Response:
We queried all variables reported as unknown to try to capture as much data as possible. Responses that remained unknown after querying were treated as missing. We added the following sentences in lines 117-119: "All responses of "unknown" were queried to facilitate optimal data capture. Responses that remained unknown after querying were treated as missing."

Results
3. *Were there any differences in baseline characteristics between observed and non-observed newborns?*

Response:
Yes, the observed population was less sick compared to the non-observed newborns. We have added Supplemental Table 1 to detail these differences and briefly summarized the differences in the results text as follows:

> "The cohort we observed with the Liveborn app was biased towards a less sick population with lower rates of Cesarean section (0.3% vs 2.2%), prematurity (12.5% vs 17.9% born at 28-36 weeks gestation), low birth weight (9.3% vs 12.8% birth weight 1500-2499g), APGAR 1min ≤ 5 (4.7% vs 7.4%), and neonatal deaths before discharge (0.9% vs 2%) compared to those not observed [Supplemental Table 1]."
>
> (Results, Lines 209-213)

UNC SCHOOL OF MEDICINE

UNC HEALTH Children's

Division of Neonatal-Perinatal Medicine
The University of North Carolina at Chapel Hill
UNC Hospitals, CB# 7596, 4th Floor, Rm N4051
Chapel Hill, NC 27599-7596
Office: 984-974-5063 • Fax: 984-974-7857

4. *It might be beneficial to see the differences between the two facilities.*

Response:
Facility 2 cares for a sicker population compared to facility 1. We have added Supplemental Table 2 to detail these differences and have briefly summarized these differences in the results text as follows:

> "Baseline characteristics by facility are notable for a sicker population at facility 2 with higher rates of Cesarean section (2.6% vs 0%), birth weight ≤ 1499 grams (4.2% vs 0.4%) and 1500-2499 grams (13.6% vs 8.8%), 28-36 weeks gestation (20.1% vs 11%), APGAR 1 min ≤ 5 (8.5% vs 3.9%), and stillbirths (2.9% vs 1.1%) along with a lower rate of neonatal transfer to another facility (0.3% vs 2.1%) [Supplemental Table 2]."
> (Results, Lines 214-218)

Of note, when our statistician was generating these two supplemental Tables, he noted a small error that the registry data was pulled from September 1, 2022 until August 30, 2023 instead of until August 31, 2023 (missing one day of data). All data now reflect that additional day of data to include a full year of the registry through August 31, 2023. We have updated the numbers in line 146, line 149, line 150, line 161, line 162, and Figure 2 accordingly. While the numbers changed slightly, the manuscript's conclusions remain the same.

Introduction

5. *Lines 65-66: I believe you can describe the seven domains fully, otherwise it looks a bit incomplete.*

Response:
We listed out the seven domains fully on lines 62-63.

Results, Table 1

6. *Text describing Table 1 is fragmented (for example, it is separated by description of Figure 3). It would be easier to read the manuscript if all the fragments were put together.*

Response:
In the published version of the manuscript, this legend will be placed directly beneath Figure 3 so that it does not separate the text describing Table 1.
