# Supplementary material for: Establishment of a neonatal resuscitation registry in the Democratic Republic of the Congo: An open cohort study
Source: PLoS One. 2025 May 23;20(5):e0324332. doi: 10.1371/journal.pone.0324332 (PMC12101705; doi:10.1371/journal.pone.0324332)
Supplement: Attachment — Submitted filename: Response to Reviewers Final.docx [file pone.0324332.s006.docx]

October 28, 2024

Dr. Ribka Amsalu
Academic Editor
PLOS ONE

Dear Dr. Amsalu,

Thank you for your further consideration of our manuscript titled “*Establishment of a neonatal resuscitation registry in the Democratic Republic of the Congo: An open cohort study” (PONE-D-24-30580).* We thank you and the reviewer for the helpful comments, and respectfully submit the final revised manuscript. Below is a point-by-point response to the reviewer’s comments.

Sincerely,

Jackie K. Patterson, MD, MPH

Associate Professor of Pediatrics

Division of Neonatal-Perinatal Medicine

University of North Carolina at Chapel Hill

**Response to Reviewer**

The current study is on a topic of relevance and general interest to the readers of the journal. I found the paper to be overall well-written and felt confident that the authors performed careful the preparation of the manuscript. However, I still have some comments and thoughts, I want to share with the authors to further improve their paper: 
 
1) Following references are missing: First two sentences of the introduction

**Response:**

We added Oza *et al.* to the first sentence of the introduction, Bellad *et al.,* Msemo *et al.,* and Versantvoort *et al.* to the second sentence of the introduction.

2) Row 52: ... critical improving / to improve .. not to improving

**Response:**

We revised per your suggestion.

3) Fig. 2: Why didn't you include newborns not crying by 60 sec in the group of interest for ILCOR?

**Response:**

The ILCOR population of interest is focused on the provision of PPV and not on those with apnea or insufficient respirations who do not receive PPV. We recognize that Figure 2 may have been confusing as we displayed those not crying by 30 seconds and those not crying by 60 seconds which are not mutually exclusive groups. We have deleted the box referring to those not crying by 60 seconds for clarity. As resuscitation algorithms emphasize initiating ventilation by 60 seconds after birth, we recommended including all newborns not crying by 30 sec.

4) I would put all footnotes next to the variable name and not next to the number to get consistency

**Response:**

We revised the footnotes in Table 1 to be next to the variable for consistency.

5) Row 194: you are not able to infer from Fig 3b on the origin of data

**Response:**

We added a reference to Table 1 in the Figure 3 legend to add transparency about the data sources.

6) Table 2: For the variable "Heart Rate -> NeoBeat -> Heart rate at initiation of PPV" did you only use data from the NeoBeat device or also from the Liveborn Observation app?

**Response:**

For Table 2, all heart rate data obtained in this cohort comes from NeoBeat. We only collected heart rate data for cases where the Liveborn Observation app was used. NeoBeat connects via Bluetooth to the Liveborn Observation app and the data is then downloaded from the Liveborn Observation app.

7) Table 2: Is there a difference between the variables "PPV -> ILCOR Supplemental -> Total duration of all episodes [min]" and "PPV -> Liveborn Observation app -> Total duration of all episodes [s]" beside from the unit?

**Response:**

Yes, the difference between “Total duration of all episodes [min]” in ILCOR supplemental and “Total duration of all episodes [s]” is the unit. Recording duration in whole units of minutes does not give enough granular data to understand gaps in care related to interruptions in PPV. Additions in the text were made in lines 268-271 to help clarify this distinction for readers.

8) Table 3: are you suggesting the adaptations as additional variables or to adapt the current recommendations 

**Response:**

In Table 3, we suggest additional variables to consider. Table 3 column header and first recommended addition were revised for clarity.

9) Please add the following points: limitations, knowledge gaps and next steps

**Response:**

We discuss the limitations of our study in the second to last paragraph of the discussion; we have clarified the content of this paragraph with a new topic sentence. We discuss knowledge gaps and next steps in the last paragraph of the discussion, and have added the following content:

“Future directions for the registry include validation of the accuracy of observational data compared to audio-video recording, and inclusion of audio-video recording data on corrective actions during PPV, competing tasks, and team communication.” (Lines 351-353)

10) Please try to include the following literature: 
DOI: 10.1016/S2214-109X(23)00302-9 Schnaubelt et al in 3^rd^ paragraph of introduction 
DOI: 10.1542/peds.2020-016915G Keenan et al in 2^nd^ paragraph of introduction 

**Response:**

We incorporated references Keenan *et al.* and Schnaubelt *et al.* in the introduction (Line 72).

11) One point of criticism is that only one of the authors is affiliated with a clinic in DRC. To create a sustainable research environment in LMICs, local researches should be encouraged and supported to do their own research projects. By including local researches in developing such projects, know-how in LMICs can be build up and even more researcher have the opportunity to find solutions in this field of interest

**Response:**

The University of North Carolina at Chapel Hill and the Kinshasa School of Public Health have had a twenty-year partnership conducting research for maternal, newborn and child health in the DRC, including through the NICHD Global Network. The registry was developed and implemented by investigators from both institutions. Drs. Ishoso and Mafuta are key members of this team and both are listed as co-authors. We noted that PLOS One allows joint first authorship; as such, we have listed Daniel Ishoso as joint first author with Amy Mackay.
